# Zearalenone Exposure Enhanced the Expression of Tumorigenesis Genes in Donkey Granulosa Cells via the *PTEN*/*PI3K*/*AKT* Signaling Pathway

**DOI:** 10.3389/fgene.2018.00293

**Published:** 2018-07-31

**Authors:** Guo-Liang Zhang, Jun-Lin Song, Chuan-Liang Ji, Yu-Long Feng, Jie Yu, Charles M. Nyachoti, Gong-She Yang

**Affiliations:** ^1^College of Animal Science and Technology, Northwest A&F University, Yangling, China; ^2^National Engineering Research Center for Gelatin-based Traditional Chinese Medicine, Dong-E-E-Jiao Co., Ltd., Liaocheng, China; ^3^Central Laboratory, Qingdao Agricultural University, Qingdao, China; ^4^Department of Animal Science, University of Manitoba, Winnipeg, MB, Canada

**Keywords:** donkey, granulosa cells, tumorigenesis, gene expression, RNA-seq

## Abstract

Zearalenone (ZEA) is a natural contaminant existing in food and feed products that exhibits a negative effect on domestic animals’ reproduction. Donkeys possess high economic value in China and are at risk of exposure to ZEA. However, few information is available on ZEA-induced toxicity and no report on toxicity in donkeys can be found in scientific literature. We investigated the biological effects of ZEA exposure on donkey granulosa cells (dGCs) by using RNA-seq analysis. ZEA at 10 and 30 μM were administered to GCs within 72 h of *in vitro* culture. ZEA at 10 μM significantly altered the tumorigenesis associated genes in dGCs. Exposure to 10 and 30 μM ZEA treatment significantly reduced mRNA expression of *PTEN*, *TGF*β, *ATM*, and *CDK2* genes, particularly, the ZEA treatment significantly increased the expression of *PI3K* and *AKT* genes. Furthermore, immunofluorescence, RT-qPCR, and Western blot analysis verified the gene expression of ZEA-exposed GCs. Collectively, these results demonstrated the deleterious effect of ZEA exposure on the induction of ovarian cancer related genes via the *PTEN*/*PI3K*/*AKT* signaling pathway in dGCs *in vitro*.

## Introduction

Zearalenone (ZEA) is a mycotoxin produced by various Fusarium fungi (Bennett and Klich, 2003) that infects grains and maize worldwide. Similar to aflatoxins (AFs), ZEA is one of the most important and widespread trichothecenes that cause extensive and recurring economic damage in cereal grains and animal feedstuffs (Escriva et al., 2015). In domestic animals, ZEA causes porcine ovarian atrophy (Vanyi et al., 1994) and equine follicular hematomas (Cortinovis et al., 2013) and exhibits significant genotoxic potential and induces DNA damage (Zhang et al., 2017a) in experimental animals (mice and rats). Owing to its estrogenic activity, ZEA could cause reproductive disorders in a wide variety of species-specific organs in animals (Poor et al., 2015). Both low and high concentrations of ZEA can cause abortion and reproductive failure in livestock (Osweiler et al., 1990; Dacasto et al., 1995; Zwierzchowski et al., 2005). Moreover, ZEA is a potential carcinogen with a possible correlation to xenoestrogens and breast cancer risk (Yu et al., 2004).

The effects of ZEA and some of its metabolites on estrous mares have only been reported in a few cases. Juhasz et al. (2001) reported the effects of 10 day low dose ZEA exposure on the reproductive system of ovulating mares fed with 7 mg purified ZEA. No effect was observed on the length of the inter-ovulatory intervals, follicular phases, and ovarian follicular activity (the maximum size and number of the ovulatory follicles, growth rate, and the initial increase in the number of large follicles) (Juhasz et al., 2001). Another study showed that feeding mares with oats that were naturally contaminated with ZEA (12 mg/kg) had no relevant effects on the cycle length and the release of reproductive hormones (Cortinovis et al., 2013). However, the authors stated that mares fed with the naturally contaminated oats had a high incidence of follicular hematomas, which did not occur in the control (Cortinovis et al., 2013). Similar to the findings in estrous pig (Zhang et al., 2017b), ZEA and its derivatives induced *in vitro* apoptosis of the granulosa cells (GCs), which were collected from estrous mares’ ovaries (Minervini et al., 2006). Hence, the authors suggested that ZEA could induce follicular atresia in domestic animals. These effects could be due to the direct interaction with ERs and 3-α/β-HSD enzymes present in the GCs and ovary, which are responsible for the synthesis or metabolism of the endogenous steroid hormones (Minervini et al., 2006).

The mechanism of ZEA toxicity is not fully understood, but ZEA is known to possess acute and chronic toxic effects in animals. Ovaries are female reproductive organs comprising follicles of varying sizes. The early stages of follicular growth depend on the development of the GCs and oocytes, which are in constant communication with each other. The development of one cell type influences the other’s compartment. During follicle development, GCs replicate, secrete hormones, and support the growth of the oocyte (Hamel et al., 2008). Previous investigations demonstrated that ZEA may alter GC’s function in swine (Ranzenigo et al., 2008; Zhang et al., 2017b). This study aims to evaluate the in vitro toxicity of 10 and 30 μM ZEA in donkey ovarian GCs through transcriptome analysis.

## Materials and Methods

### Reagents

Zearalenone was purchased from Sigma Company (St. Louis, MO, United States). Stock solutions of ZEA were fixed by dissolving ZEA in dimethyl sulfoxide (DMSO). DMSO (D12345), fetal bovine serum (FBS; 10100147), M-199 medium (11150-059), penicillin and streptomycin were procured from Gibco Company (Carlsbad, CA, United States).

### Animals

The mature donkeys’ ovaries used in the experiment were obtained from the Dong E Donkey Production Company (Qingdao, Shandong, China). The ovaries were collected from the slaughterhouse of the company and maintained at 32–35°C for the isolation of GCs. All procedures of animal handling in this study were reviewed and approved by the Ethical Committee (Agreement No. 2017-18) of Qingdao Agricultural University.

### Isolation and Culture of dGCs

Donkey GCs (dGCs) were aspirated from the antral follicles using a 10 ml syringe (Zhu et al., 2012). Standing for 15–18 min, the dGCs were centrifuged at 300 *g* for 5 min in accordance with previous report (Qin et al., 2015). Then the dGCs were cultured in DMEM medium (HyClone, SH30022.01, Beijing, China) supplement with 10% FBS (10099-141, Gibco, Australia) and 1% penicillin-streptomycin (Hyclone, SV30010) in incubator with 5% CO_2_ at 37°C (Duda et al., 2011).

The primary GCs were passaged after culture 48–36 h. To avoid the stress of passage response, drug exposure was performed until 12 h later. The GCs were inoculation in a 6 cm petri dish (Corning, 430166, United States) at a density of 1 × 10^6^ cells. ZEA was added to the cultured medium at final concentrations of the 10 or 30 μM, then the cells were incubated with ZEA for 72 h. The control and 10 μM ZEA group added the same dose of DMSO to 30 μM ZEA group for accuracy.

### Immunofluorescence and Cell Counting

The GCs were collected and fixed in 4% paraformaldehyde for 2 h, then heated at 42°C for 2 h, finally attached onto a poly-lysine coated slide. For immunofluorescence, the sections were blocked with the BDT (10% goat serum in TBS, 3% BSA) for 35 min, and then incubated overnight with primary antibodies at 4°C (**Supplementary Table S1**). The sections were then incubated with Cy3/FITC-conjugated goat anti-rabbit secondary antibody (Beyotime, A0208, Nantong, China) at a dilution of 1:50 at 37°C for 1.5 h. Finally, the sections were incubated with Hoechst33342 (Beyotime, C1022) to visualize nuclei of GCs for 3 min at room temperature. The immunosignal was detected using a fluorescence microscope (Olympus, XB51, Japan), the images were captured and analyzed in accordance with cellSens Standard.

### TUNEL Staining

TUNEL BrightRed Apoptosis Detection Kit was utilized to evaluate the GCs apoptosis (Vazyme, A11302, Nanjing, China). Briefly, the GCs were fixed with 4% paraformaldehyde for 2 h after ZEA treatment. Observation under fluorescence microscopy was carried out after TUNEL reaction following manufacturer’s recommendations [60 min at 37°C without light, and DNA staining with Hoechst 33342 (Beyotime, C1022) incubation at room temperature for 3 min]. The TUNEL positive cells were detected under the fluorescence microscope (Olympus, XB51). To analyze TUNEL positive cell ratio, more than 2,000 cells were counted in each group, and each group included three biological replicates at least.

### RNA Extraction, Reverse Transcription, and RNA-seq

Total RNA was extracted from the cultured GCs by the use of an RNAprep pure MicroKit in line with the manufacturer’s instruction (Aidlab, RN07, Beijing, China). And the first-strand cDNA acquisition was utilized a cDNA Synthesis Kit (TransGen, AT311-03, Beijing, China) with reference to previous study (Pan et al., 2011). The reaction program was setup at: 42°C for 15 min, 85°C for 30 s, and 4°C for cooling. Then, RNA sequencing was performed with Hiseq 4000 platform by Novogene (Beijing, China). Three biological replicates were manipulated in each group. Primary RNA-seq data were uploaded to the SRA repository. The SRA index number was SRP139976. The RNA-seq data matrix used for DESeq2 analysis were uploaded to the GEO repository and the GEO accession number was GSE116950^1^.

### Data Preprocessing and Identification of Differentially Expressed Genes

R Bioconductor/DESeq2 package was applied to analyze the dGCs groups (control, 10 and 30 μM ZEA) to identify the difference of gene expression. DESeq2, the negative binomial was applied as the reference distribution and taken own normalization approach for raw counts in differential expression analysis. In other methods to avoid possible biases, the data of differential expression analysis has to be previously normalized (Benjamini et al., 2001; Luo and Brouwer, 2013). Because the design of sequencing contains biological replicates in each group, log2|fold change| is not setup as the filter condition. So *p*_adj_ < 0.01 was really considered as statistical significance.

### KEGG Enrichment Analysis

R Bioconductor/clusterProfiler package was applied to analyze functional profiles (KEGG) of differentially expressed genes (DEGs) (Yu et al., 2012). Furthermore, R Bioconductor/Pathview package was applied to visualize KEGG enrichment results. According to the DEGs log2|fold change| value shows enrichment signaling pathway active status. The p-value adjusted used the Benjamini & Hochberg (BH) method (Luo and Brouwer, 2013). Still, the *p*_adj_ < 0.05 was considered as statistical significance.

### Gene Set Enrichment Analysis

Gene set enrichment analysis (GSEA) does not need to specify a clearly differential expression of gene threshold, the algorithm performs GSEA based on overall trend of the gene raw read count. The file has to be made suitable for GSEA software analysis with R script (Subramanian et al., 2005). The data for GSEA analysis contained nine samples within three groups. The GSEA software option “Collapse dataset to gene symbols” parameters was “false” and option “Permutation type” parameters were “gene set.” Other parameters were set as default as software manual references. The GSEA gene set with FDR *q*-value <0.05 were defined as significant difference.

### Protein–Protein Interaction Network Construction and Modules Mining

Search Tool for the Retrieval of Interacting Genes/Proteins^2^ (STRING) is a database of protein–protein interaction (PPI). This database contains the direct and physically related interactions between known and predicted protein and genes. The R Bioconductor/STRINGdb was applied for PPI of interested DEGs (Franceschini et al., 2013). The Cytoscape software was applied to visualize PPI results (Shannon et al., 2003).

### Quantitative Real-Time PCR

Total RNA and cDNA reverse transcription was extracted as described above. Quantitative real-time PCR (RT-qPCR) was performed using Light-Cycler^®^ 480 SYBR Green I Master Kit (Roche, Germany) on LightCycler 480 real-time PCR instrument. The RT-qPCR reaction was set as: 10 min at 95°C, followed by 45 cycles of 95°C for 10 s, 60°C for 30 s, 72°C for 30 s, and cooling step at 4°C. Quantitative RT-PCR primers are listed in **Supplementary Table S2** and the primer efficiency in the **Supplementary Figure S1**. The standard curve gene *GAPDH* in dGCs was used as the reference to normalize the related genes’ mRNA expression. The difference multiples = 2^-ΔΔCT^ method was employed for relative quantification of PCR. Each gene was expressed as the mean ± standard deviation (SD), which was calculated from independent biological replicates at least three times.

### Western Blotting

Protein lysates isolated from dGCs were used for western blotting according to the standard protocol (Zhang et al., 2010; Chao et al., 2012). Proteins from each ZEA treatment were separated by 10% SDS-PAGE, then transferred onto the PVDF membranes. After, the membranes were incubated with primary antibodies (**Supplementary Table S1**) at 4°C overnight. Then the membranes were incubated with secondary antibodies (Beyotime, A0208) at 37°C for 2 h in TBST after rinsing three times with TBST. The final detection of related genes was carried out by AlphaImager^®^ HP (ProteinSimple, 92-13824-00, United States). The band intensity was quantified using *GAPDH* as internal control and measured with IPWIN software.

### Statistical Methods

Data are presented by mean ± SD. Different effects between the control and ZEA treatment groups of donkey was statistically determined by One-way ANOVA for multiple comparisons. All analyses were conducted using Graph-Pad Prism analysis software (San Diego, CA, United States). All experiments were repeated at least three times unless otherwise noted. Results were considered statistically significant at *p*-value <0.05.

## Results

### The Apoptosis and Apoptosis-Related Gene Expression of dGCs Exposed to ZEA

**FIGURE 1:**
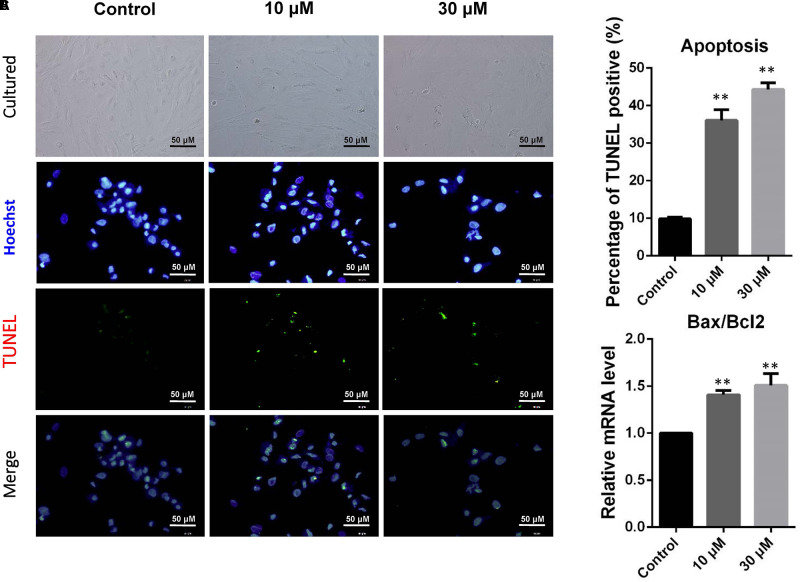
Zearalenone exposure increasing apoptosis and inducing the apoptosis-related gene mRNA abundance in cultured GCs. GCs were stained blue with Hoechst33342 Solution. TUNEL assay was performed using immunostaining. **(A)** Immunofluorescent staining of TUNEL and Hoechst33342 of GCs. Bar indicates 50 μm. **(B)** The percentages of TUNEL positive GCs. **(C)** The mRNA levels of *BAX/BCL2* in cultured GCs exposed to ZEA, and the control determined by Q-PCR. The results are presented as mean ± SD. All experiments were repeated at least three times. ^∗^*P* < 0.05; ^∗∗^*P* < 0.01.

Donkey granulosa cells were cultured *in vitro* and exposed to 10 or 30 μM ZEA for 72 h (**Figure 1A**). The percentages of TUNEL positive dGCs significantly increased as a result of exposure of ZEA (10 μM: 36.04 ± 1.52%; 30 μM: 44.30 ± 1.33%) compared to that of the control (0 μM: 9.83 ± 0.21%; *P* < 0.01; **Figure 1B**). As shown in **Figure 1C**, the ratios of *BAX/BCL2* mRNA expression significantly increased in 10 and 30 μM ZEA exposed dGCs.

**FIGURE 2:**
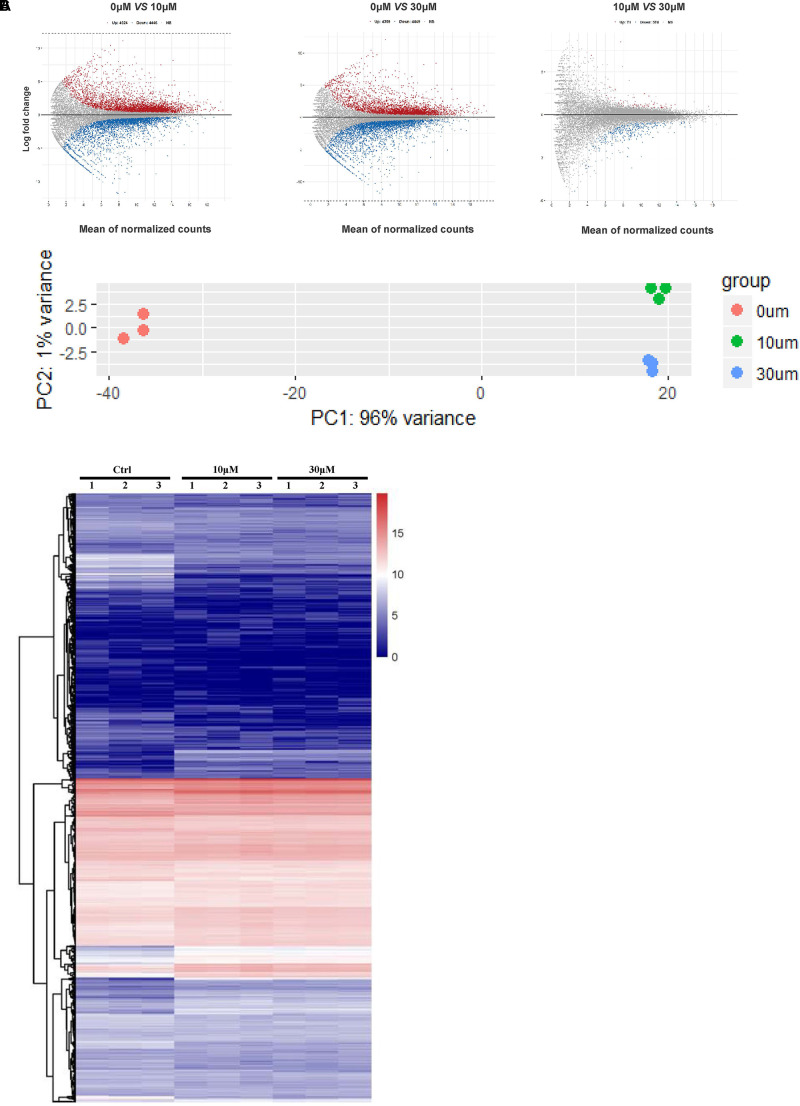
The GCs gene expression profiling after ZEA treatment. **(A)** Scatterplot of gene expression after ZEA treatment. Control group vs. 10 μM ZEA-treatment group, control group vs. 30 μM ZEA-treatment group, 10 μM ZEA-treatment group vs. 30 μM ZEA-treatment group. *Red* and *blue plot* represents genes expressed differently. **(B)** The nine samples shown in the 2D plane spanned by the first three principal components. **(C)** Heatmap indicate the group difference of DEGs in the 10 and 30 μM ZEA-treated groups compared with the control group, and the repeatability within each group. The results are presented as mean ± SD. All experiments were repeated at three times.

**FIGURE 3:**
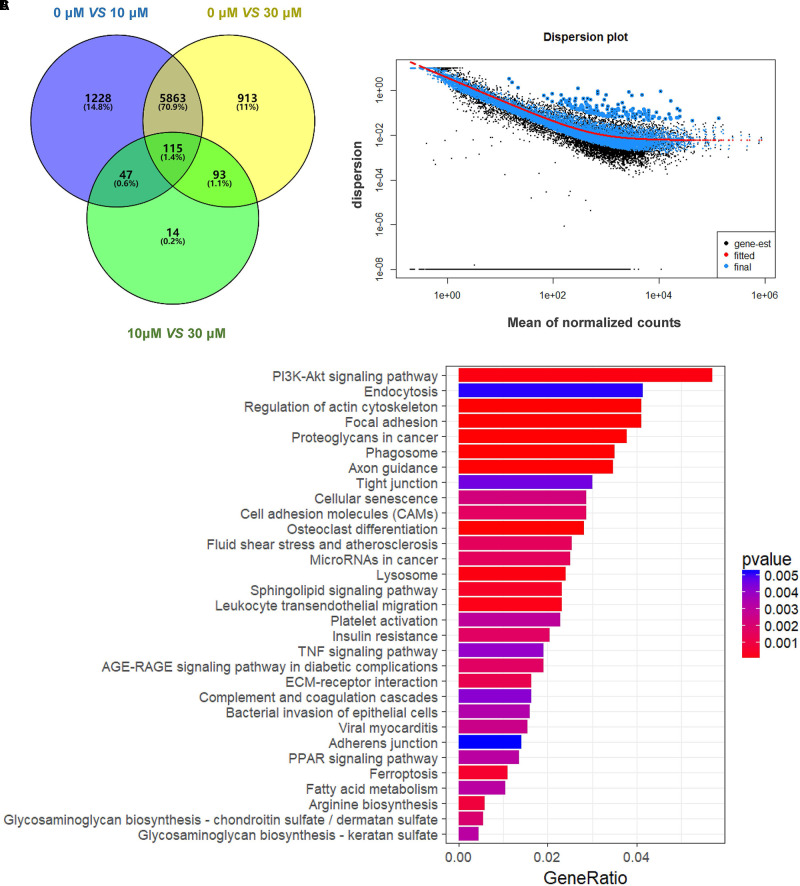
Gene expression of RNA-seq analysis in granulosa cells between control and ZEA-treated groups. **(A)** Venn diagram showed that the different expression of 14,506 genes in control and ZEA-treated groups. **(B)** Dispersion plot. The dispersion estimate plot shows the gene-wise estimates (*black*), the fitted values (*red*), and the final maximum a posteriori estimates used in testing (*blue*). **(C)** KEGG biological processes involving DEGs in ZEA-treatment GCs.

We performed RNA-seq to verify the effect of ZEA exposure on dGCs. Based on the criterion FDR <0.05, 14,506 DEGs was observed between the control and the ZEA-treated dGCs (**Figure 2A**). A total of 7,253 and 6,984 DEGs were noted between the control and 10 and 30 μM ZEA treatment groups, respectively. A total of 269 DEGs were observed between the 10 and 30 μM ZEA treatment groups. In this study, the DEGs between the control and ZEA treatment groups and among the treatment groups were obtained from: 0 μM vs. 10 or 30 μM and 10 vs. 30 μM ZEA group, respectively (**Figure 3A**).

We obtained three RNA-seq replicates for the 0, 10, and 30 μM ZEA-treated dGCs. The variation of the replicates from the control and ZEA treatment groups are shown in **Figure 2B**. A heat map was drawn from the DEG results (**Figure 2C**). In this study, we chose the DEGs from the 0 μM vs. 10 and 30 μM groups.

To explore the potential mechanism of ZEA exposure in dGCs, the STRING database was applied in annotating functional interactions DEGs between the control and ZEA-treatment group. Also PPI networks was visualized by Cytoscape (**Supplementary Figures S2A**). The center nodes of the networks, some interesting genes, were observed, such as *PTEN*, *AKT*, *ATM*, *TGF*β, *PI3K*, *CCND2, HEY2, CDK2*, and *FDX1*. In addition, the cBioPortal was used to provide visualization, analysis of DEGs related to the ovarian cancer from large-scale cancer genomics data sets (**Supplementary Figures S2C**, **S3**).

### DEGs Involved in KEGG Pathways

In order to attain functional insights of DEGs, the R package of clusterProfiler was carried out to establish extremely affected KEGG pathways. The Venn diagram were constructed by the results of DEGs (**Figure 3A**). A total of 5,863 DEGs containing in control and 30 μM ZEA groups were quantified (**Figure 3B**). The DEGs were significantly enriched in the *PI3K*-*AKT* signaling pathway (Count = 125, *p*_adj_ = 0.0049), endocytosis (Count = 91, *p*_adj_ = 0.049), regulation of actin cytoskeleton (Count = 90, *p*_adj_ = 0.0001), and proteoglycans in cancer (Count = 83, *p*_adj_ = 0.0009) in ZEA treated dGCs (**Figure 3C**).

**FIGURE 4:**
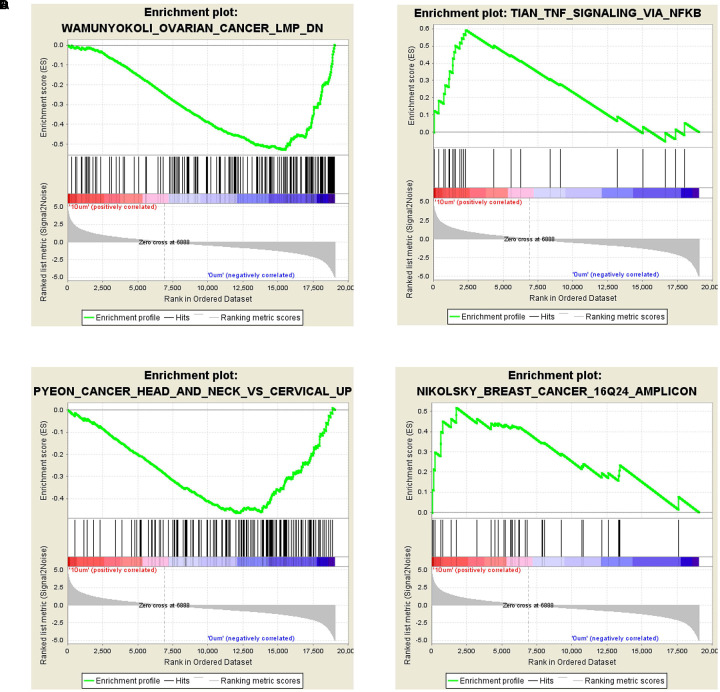
Differentially expressed genes in GSEA analysis. **(A,B)** GSEA for GCs treated with 10 μM ZEA. **(C,D)** GSEA for GCs treated with 30 μM ZEA.

The GSEA analysis showed that *OVARIAN_CANCER_LMP* and *TNF_SIGNALING_VIA_NFKB* gene set was enriched by 10 μM ZEA vs. the control of dGCs (**Figures 4A,B**), while *CANCER_HEAD_AND_NECK_VS_CERVICAL* and *BREAST_CANCER_16Q24_AMPLICON* gene set was enriched by the 30 μM ZEA vs. the control in dGCs (**Figures 4C,D**), respectively. The GSEA enrichment plot revealed a regulated tendency for most of enriched genes in ZEA-treatment compared with the control. The GSEA analysis also verified the KEGG analysis.

In addition, six genes were identified by performing the KEGG and GSEA pathway analysis in ZEA treatment, the *PI3K* and *AKT* genes were involved in *PI3K*-*AKT* signaling pathway of dGCs upregulating. DGCs in ZEA treatment, another four genes involved in anti-oncogene process downregulating were also identified, such as *PTEN*, *TGF*β, *CDK2*, and *ATM*. Finally, *CCND2, CDK6, TNF*, and *TP53* genes in treatment dGCs, were identified involving in cancer process either upregulation or downregulation.

### Specific Impact of ZEA Exposure to dGCs

**FIGURE 5:**
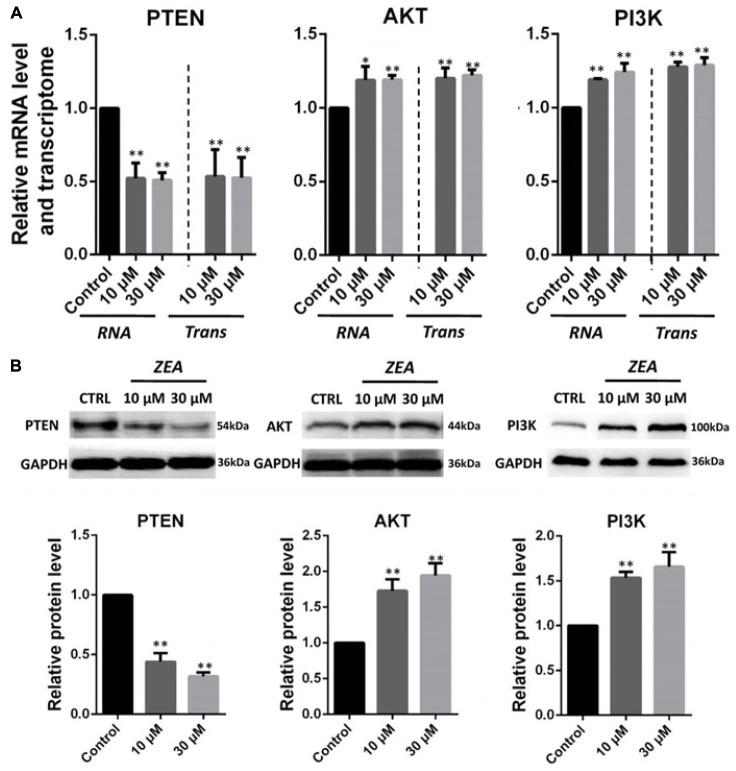
Zearalenone exposure affecting mRNA and protein abundance of *PTEN*-related genes in cultured donkey GCs. **(A)** Quantitative RT-PCR for *PTEN*, *AKT*, and *PI3K* transcription factors. The mRNA levels of all genes were normalized to GCs *GAPDH* gene. **(B)** Protein levels of *PTEN/GAPDH*, *AKT/GAPDH*, and *PI3K/GAPDH* by Western blotting. The protein levels were normalized to *GAPDH*. The results are presented as mean ± SD. All experiments were repeated at least three times. ^∗^*P* < 0.05; ^∗∗^*P* < 0.01.

**FIGURE 6:**
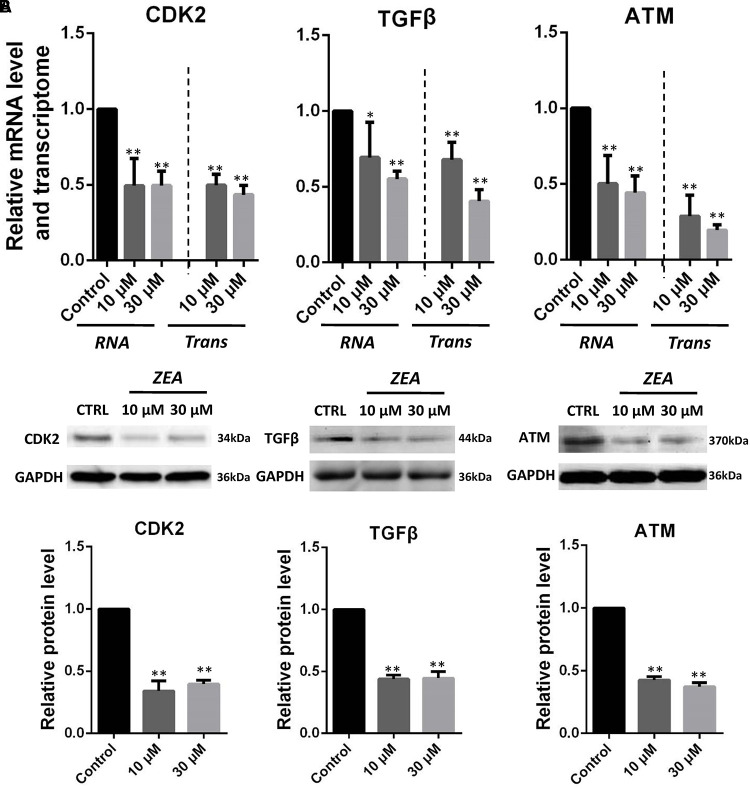
Zearalenone exposure affecting mRNA and protein abundance of tumorigenesis related genes in cultured GCs. **(A)** Quantitative RT-PCR for *CDK2*, *TGF*β, and *ATM* transcription factors. The mRNA levels of all genes were normalized to GCs *GAPDH* gene. **(B)** Protein levels of *CDK2*/*GAPDH TGF*β/*GAPDH*, and *ATM*/*GAPDH* by Western blotting. The protein levels were normalized to *GAPDH*. The results are presented as mean ± SD. All experiments were repeated at least three times. ^∗^*P* < 0.05; ^∗∗^*P* < 0.01.

**FIGURE 7:**
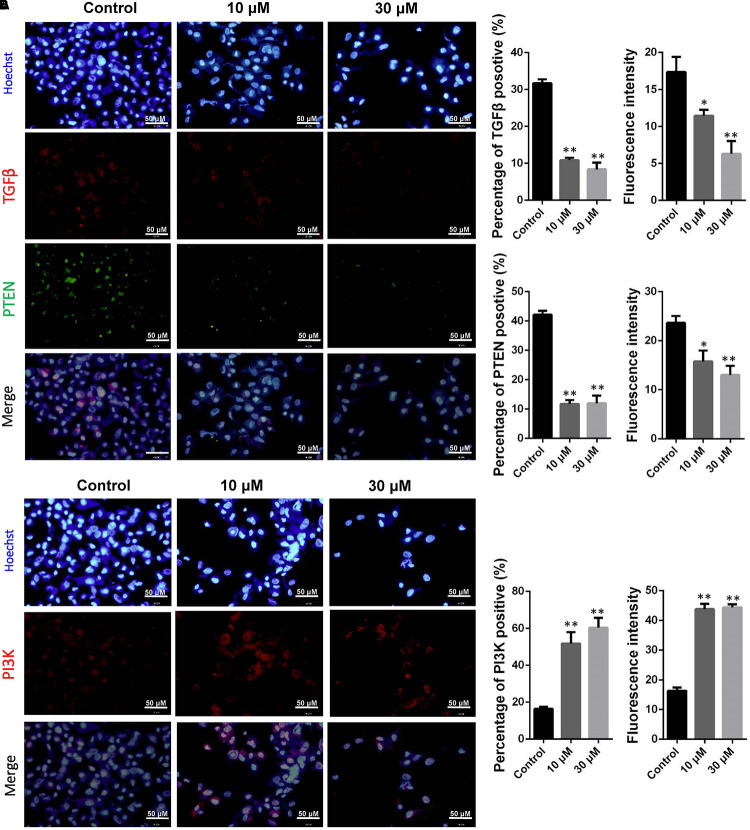
Immunofluorescence assay examining the expression of phosphor-*PTEN*, *TGF*β **(A)**, and *PI3K* **(B)** proteins. The fluorescence intensity and percentages of positive cells were analyzed, respectively. Bar indicates 50 μm. Data are presented as means ± SD. ^∗^*P* < 0.05; ^∗∗^*P* < 0.01.

It is hypothesis that 10 and 30 μM ZEA exposure may affect the proliferation and carcinogenesis of dGCs (**Figure 4**). RT-qPCR was conducted to verify the hypothesis by evaluating the expression of different transcript together with enzymes in the pathway of dGCs between the control and ZEA treatments. RT-qPCR analyses indicated that 10 and 30 μM ZEA exposure significantly downregulated mRNA abundance of *PTEN* while upregulated *PI3K* and *AKT* genes in dGCs (*P* < 0.05 or *P* < 0.01; **Figure 5A**). As illustrated in **Figure 5B**, ZEA-treated dGCs exhibited lower protein levels of *PTEN* but higher protein levels of *PI3K* and *AKT* compared with that of the control dGCs (*P* < 0.05 or *P* < 0.01). Moreover, 10 and 30 μM ZEA exposure significantly down-regulated the mRNA abundance and protein levels of *CDK2*, *TGF*β, and *ATM* genes than the control dGCs (*P* < 0.05 or *P* < 0.01; **Figures 6A,B**). Interestingly, the ZEA treatments significantly decreased the number of *PTEN* and *TGF*β but significantly increased the number of *PI3K* immunofluorescence positive dGCs, in comparison to the control (**Figures 7A,B**).

## Discussion

Numerous studies have proven ZEA’s cytotoxic effect on the reproductive (Kiang et al., 1978; Mehmood et al., 2000; Nikov et al., 2000), immune (Abbes et al., 2006a,b; Luongo et al., 2006), and endocrine (Mueller et al., 2004) systems, as well as on heredity (Kouadio et al., 2005). ZEA indirectly affects mammalian fertility by impairing the formation of primordial follicle (Zhang et al., 2017a), thereby causing changes in gene expression; it also induces DNA damage in ovarian GCs (Zhang et al., 2017b). Not much information is available on ZEA-induced alteration in dGC proliferation and development and impairment of mammalian fertility. This research is the first to describe the GC transcriptomes of donkeys.

The RNA-seq results showed that exposure to 10 μM ZEA significantly transformed the mRNA expression of thousands of genes in dGCs. In particular, *PTEN* genes were downregulated, suggesting that ZEA exposure is involved with the cancer-related *PTEN* signaling pathway. The tumor suppressor gene, *PTEN*, plays an essential role in cell growth, survival, and tumor formation. Decrease in *PTEN* expression, either at the protein or mRNA level, has been associated with many primary malignancies, including ovarian cancer (Kechagioglou et al., 2014). Hence, the downregulation of *PTEN* is a hallmark of tumors. The *PI3K*-*AKT* pathway is one of the two major signaling pathways that have been identified as important in cancer. Through phosphorylation, *PI3K*-*AKT* inhibits the activity of proapoptotic members while activating anti-apoptotic members. The reduction in *PTEN* expression indirectly stimulates *PI3K*-*AKT* activity, thereby contributing to oncogenesis in mammals (Zhang et al., 2017c). Our studies showed that exposure to 10 μM ZEA significantly upregulated the *PI3K*-*AKT* gene expression in the dGCs, implying that exposure to low concentrations of ZEA (10 μM) might increase the donkey’s risk for ovarian cancer via the *PTEN*/*PI3K*/*AKT* pathway by suppressing the expression of antitumor genes or by activating the expression of cancer-causing genes (Zhang et al., 2017c). Moreover, it was reported that decreased *TGF*β-mediated signaling might predispose an individual to develop cancer (Pasche, 2001; Galliher et al., 2006; Jin et al., 2017). Previous studies have assessed the association between *TGF*β and risk for various forms of cancer, and several meta-analyses have demonstrated that *TGF*β is associated with risk for ovarian cancer (Akhurst and Derynck, 2001; Nilsson and Skinner, 2002; de la Cruz-Merino et al., 2009). Interestingly, *TGF*β expression in the ZEA treatment samples was significantly decreased, indicating an increased risk for ovarian cancer. The direct effect of *TGF*β is not solely responsible for influencing tumor behavior (Hagedorn et al., 2001; Tuxhorn et al., 2002). Lack of *TGF*β in fibroblasts can result in mammary gland tumor progression (Bhowmick et al., 2004; Matise et al., 2012).

Bioinformatics analysis confirmed that ataxia-telangiectasia mutated (*ATM*) and cyclin-dependent kinase 2 (*CDK2*) genes were affected by exposure to ZEA. A previous study showed that the *ATM* gene plays an essential role in DNA double-strand breaks (DSBs) repair (Kurz and Lees-Miller, 2004). These DSBs if left unchecked can result in the development of cancer (Weber and Ryan, 2015). Several studies showed that suppression of *ATM* is associated with a variety of tumors (Weber and Ryan, 2015). Here, we provide evidence that exposure to 10 μM ZEA significantly decreased *ATM* gene expression, suggesting a common mechanism of action in donkeys. Other studies revealed that cell cycle dysregulation, resulting in uncontrolled proliferation, is also a hallmark of cancer (Vijayaraghavan et al., 2017). The *CDK* family is composed of proteins associated with cell cycle regulation and is frequently mutated or overexpressed in ovarian cancer. Deregulation of the *CDK2/4/6* signaling pathway is among the most common aberrations found in ovarian cancer (D’Andrilli et al., 2004). Interestingly, exposure to 10 μM ZEA significantly downregulated the expression of *CDK2* genes, which regulate the cell cycle and are involved in ovarian cancer. Finally, exposure to ZEA increased the dGCs’ apoptosis rate and elevated the expression of the *BAX/BCL2* genes.

## Conclusion

This research is the first study to investigate ZEA-induced impairment of dGCs. ZEA (10 or 30 μM), a potentially carcinogenic substance, can directly cause tumorigenesis and in vitro apoptosis of dGCs. This study developed an innovative, integrated, and low-cost approach to study GC exposure to ZEA. ZEA disrupts the endocrine and reproductive performance of domestic animals, and this study may help elucidate the mechanism of ZEA toxicity.

## Ethics Statement

All donkeys were treated humanely during slaughter (No. 11002009000012, production license number: SCXK (Qingdao): 2012–0766, Dongeejiao, Qingdao, China). The procedures of animal handling in this study were reviewed and approved by the Ethical Committee of Qingdao Agricultural University (Protocol No. 2017-018).

## Author Contributions

G-LZ and G-SY designed the study and wrote the manuscript. J-LS, C-LJ, Y-LF, JY, and CN conducted the experiments and analyzed the data.

## Conflict of Interest Statement

C-LJ, Y-LF, and JY were employed by company Dong-E-E-Jiao Co., Ltd. The remaining authors declare that the research was conducted in the absence of any commercial or financial relationships that could be construed as a potential conflict of interest.

## Footnotes

1
https://www.ncbi.nlm.nih.gov/geo/query/acc.cgi?acc=GSE116950

2
https://string-db.org/

## Supplementary Material

FIGURE S1 The standard curve and primer efficiency of all Q-PCR primers. **(A–F)** represent the standard curve and primer efficiency of *PTEN*, *AKT*, *PI3K*, *CDK2*, *TGF*β, and *ATM* genes.

FIGURE S2 Protein–protein interaction (PPI) network based on the STRING database to annotate functional interactions between DEGs in control and 10 μM ZEA-treatment groups **(A)**, in control and 30 μM ZEA-treatment groups **(B)**. Cancer type detailed summary of DEGs related to the ovarian cancer **(C)**. The node degree ≥20 was selected as the threshold.

FIGURE S3 Visualization of DEGs related to the ovarian cancer from large-scale cancer genomics data sets. The node degree ≥20 was selected as the threshold.

TABLE S1 Primary antibodies.

TABLE S2 Primers used for quantitative-PCR.
