# Corrigendum: Zearalenone exposure enhanced the expression of tumorigenesis genes in donkey granulosa cells via the *PTEN/PI3K/AKT* signaling pathway

**DOI:** 10.3389/fgene.2025.1564143

**Published:** 2025-02-17

**Authors:** Guo-Liang Zhang, Jun-Lin Song, Chuan-Liang Ji, Yu-Long Feng, Jie Yu, Charles M. Nyachoti, Gong-She Yang

**Affiliations:** ^1^ College of Animal Science and Technology, Northwest A&F University, Yangling, China; ^2^ National Engineering Research Center for Gelatin-based Traditional Chinese Medicine, Dong-E-E-Jiao Co., Ltd., Liaocheng, China; ^3^ Central Laboratory, Qingdao Agricultural University, Qingdao, China; ^4^ Department of Animal Science, University of Manitoba, Winnipeg, MB, Canada

**Keywords:** donkey, granulosa cells, tumorigenesis, gene expression, RNA-seq

In the published article, there was an error in Figure 5B as published. The Western blot picture of the PI3K gene is incorrect**.** The corrected Figure 5B and its caption Figure 5 appear below.

**FIGURE 5:**
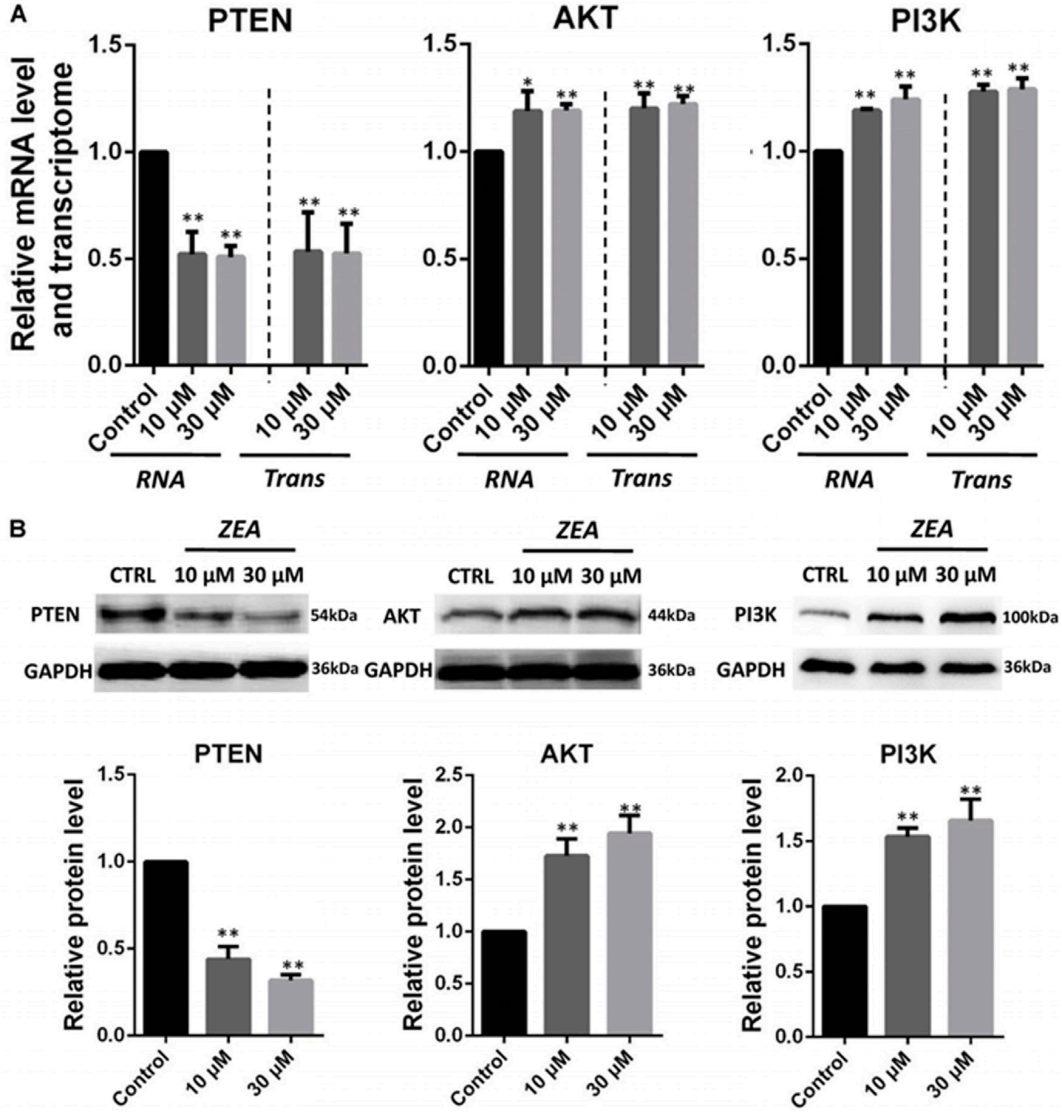
Zearalenone exposure affecting mRNA and protein abundance of tumorigenesis related genes in cultured GCs. **(A)** Quantitative RT-PCR for *CDK2*, *TGFβ*, and *ATM* transcription factors. The mRNA levels of all genes were normalized to GCs *GAPDH* gene. **(B)** Protein levels of *CDK2/GAPDH TGFβ/GAPDH*, and *ATM/GAPDH* by Western blotting. The protein levels were normalized to *GAPDH*. The results are presented as mean ± SD. All experiments were repeated at least three times. *P < 0.05; **P < 0.01.

The authors apologize for this error and state that this does not change the scientific conclusions of the article in any way. The original article has been updated.

