# Supplementary figures and images for: Zearalenone Exposure Enhanced the Expression of Tumorigenesis Genes in Donkey Granulosa Cells via the PTEN/PI3K/AKT Signaling Pathway
Source: Front Genet. 2018 Jul 31;9:293. doi: 10.3389/fgene.2018.00293 (PMC6079390; doi:10.3389/fgene.2018.00293)

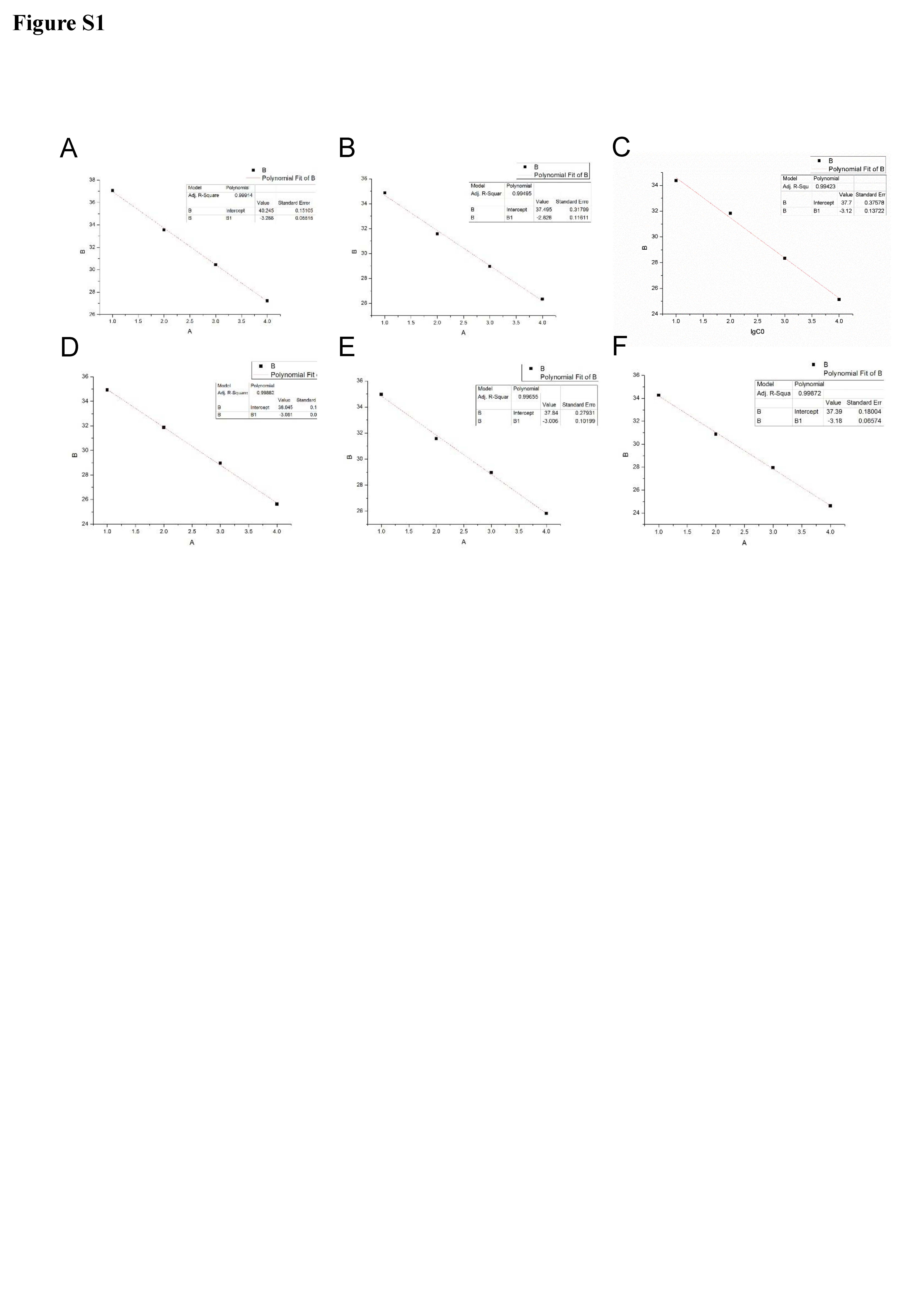

Supplement: FIGURE S1 — The standard curve and primer efficiency of all Q-PCR primers. (A–F) represent the standard curve and primer efficiency of PTEN, AKT, PI3K, CDK2, TGFβ, and ATM genes. [file Image_1.jpeg]

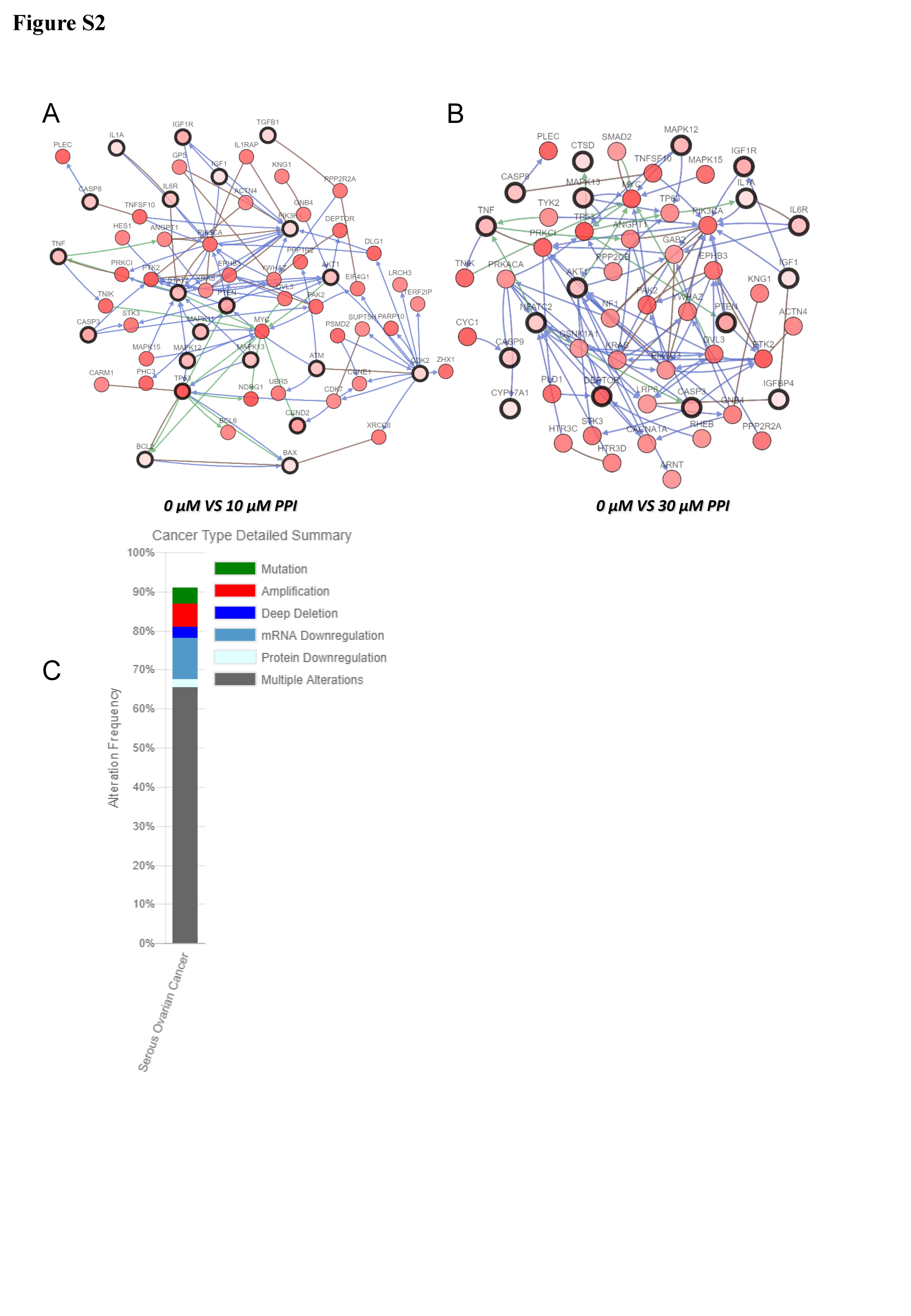

Supplement: FIGURE S2 — Protein–protein interaction (PPI) network based on the STRING database to annotate functional interactions between DEGs in control and 10 μM ZEA-treatment groups (A), in control and 30 μM ZEA-treatment groups (B). Cancer type detailed summary of DEGs related to the ovarian cancer (C). The node degree ≥20 was selected as the threshold. [file Image_2.jpeg]

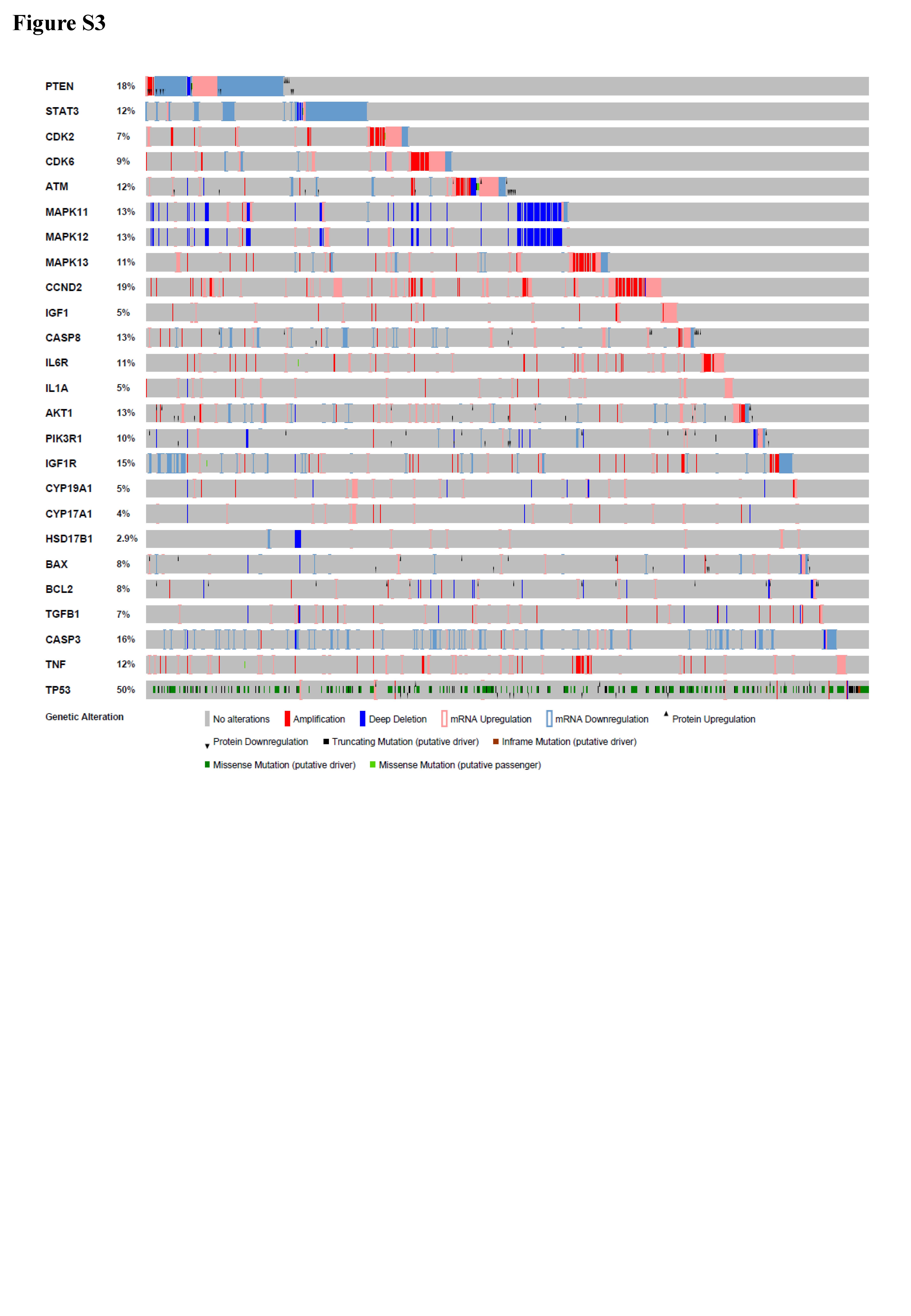

Supplement: FIGURE S3 — Visualization of DEGs related to the ovarian cancer from large-scale cancer genomics data sets. The node degree ≥20 was selected as the threshold. [file Image_3.jpeg]
