# Supplementary material for: Zearalenone Exposure Enhanced the Expression of Tumorigenesis Genes in Donkey Granulosa Cells via the PTEN/PI3K/AKT Signaling Pathway
Source: Front Genet. 2018 Jul 31;9:293. doi: 10.3389/fgene.2018.00293 (PMC6079390; doi:10.3389/fgene.2018.00293)
Supplement: TABLE S1 — Primary antibodies. [file Table_1.docx]

**Table S1** Primary Antibodies

| Proteins | Article Number | Producer | Origin |
| --- | --- | --- | --- |
| PTEN | D261095 | Sangon | Shanghai, China |
| CDK2 | D120395 | Sangon | Shanghai, China |
| GAPDH | ab70699 | Abcam | Hong Kong, China |
| AKT | ab18785 | Abcam | Hong Kong, China |
| PI3K | ab232997 | Abcam | Hong Kong, China |
| TGFβ | ab220084 | Abcam | Hong Kong, China |
| ATM | bs1370R | Bioss | Beijing, China |

**Table S2** Primers Used for Quantitative-PCR

| Genes | Sequences of primers | Products(bp) | Genbank |
| --- | --- | --- | --- |
| *PTEN* | F:5＇- AAGGGACGAACTGGTGTAATG -3＇ | 136 | XM_014864359.1 |
|  | R:5＇- GCCTCTGACTGGGAATAGTTAC -3＇ |  |  |
| *AKT* | F:5＇- GTGGTCATGTACGAGATGGTG -3＇ | 81 | XM_014854427.1 |
|  | R:5＇- CATGAGGATGAGCTCGAAGAG -3＇ |  |  |
| *PI3K* | F:5＇- GTCTGGCCTAATGTAGAAGCAG -3＇ | 149 | XM_014850184.1 |
|  | R:5＇- TCCAGCCAATCTACTTTCACC -3＇ |  |  |
| *CDK2* | F:5＇- CGAGACCTCAAACCTCAGAATC -3＇ | 150 | XM_014857244.1 |
|  | R:5＇- CAAAAGGATTTCAGGTGCTCG -3＇ |  |  |
| *TGFβ* | F:5＇- AAGGTGTGGACAGTGAAGATG -3＇ | 89 | XM_014840877.1 |
|  | R:5＇- GAGGATTAGATGAGGGTTGTGG -3＇ |  |  |
| *ATM* | F:5＇- ACACTAGCCCGAACTTTCAAG -3＇ | 130 | XM_014860766.1 |
|  | R:5＇- CTTTTGCCCAGAATACTTGCG -3＇ |  |  |
| *BAX* | F:5＇- GAGCTGGACAGTAACATGGAG -3＇ | 147 | XM_014852261.1 |
|  | R:5＇- GGCAAAGTAGAAAAGGGCAAC -3＇ |  |  |
| *BCL-2* | F:5＇- CCTGTGGATGACTGAATACCTG -3＇ | 120 | XM_014843802.1 |
|  | R:5＇- CAGGAGAAATCAAACAGCGG -3＇ |  |  |
| *GAPDH* | F:5＇- GAAAGCTGCCAAATACGATGAG -3＇ | 136 | XM_014866500.1 |
|  | R:5＇- GAAGGTGGAAGAGTGGATGTC -3＇ |  |  |
